# Leveraging AI and Machine Vision in Telephysiotherapy for Promoting Physical Activity and Reducing Sedentary Behavior: Scoping Review

**DOI:** 10.2196/80459

**Published:** 2026-10-15

**Authors:** Liva Redliha, Guna Semjonova, Liva Araka

**Affiliations:** 1 Department of Rehabilitation, Faculty of Health and Sports Sciences, Riga Stradiņš University, Dzirciema Street 16, Riga, LV-1007, Latvia, 371 67 409 219; 2 Faculty of Medicine, Riga Stradiņš University, Riga, Latvia

**Keywords:** healthy adults, artificial intelligence, AI, machine vision, telephysiotherapy, machine learning

## Abstract

**Background:**

Globally, sedentary behavior is a leading risk factor for noncommunicable diseases and premature death. Although telerehabilitation has emerged as a promising method to increase access to physical therapy, the potential of artificial intelligence (AI) and machine vision technologies to objectively monitor and promote physical activity (PA) in home settings remains underexplored.

**Objective:**

This scoping review aimed to map and systematize current evidence on the use of AI and machine vision in telephysiotherapy to promote PA and reduce sedentary behavior among healthy adults.

**Methods:**

The review followed the Joanna Briggs Institute scoping review methodology and was reported according to the PRISMA-ScR (Preferred Reporting Items for Systematic Reviews and Meta-Analyses Extension for Scoping Reviews) guidelines. The PCC (population, concepts, and context) framework defined participants as healthy adults, the concept as AI or machine learning (ML) and machine vision technologies, and the context as remote, home-based telephysiotherapy for objective PA monitoring and/or outcome measurement in interventions aimed at promoting daily activity. Searches were conducted in PubMed, ProQuest, Science Direct, and Wiley Online Library for peer-reviewed primary studies published between 2016 and 2024. Inclusion and exclusion criteria were applied systematically using the Rayyan platform.

**Results:**

Of 7253 initial records, 4 studies met the inclusion criteria. Identified digital solutions comprised ML algorithms (eg, extreme gradient boosting and hybrid classifiers), deep learning models such as graph convolutional networks, video-based center-of-pressure assessments using RGB cameras, and ontology-driven semantic data structuring for personalization. The most frequently studied technologies combined wearable sensors with ML-based behavior change frameworks to assess usability and prototype feasibility. However, none of the studies demonstrated statistically significant improvements in reducing sedentary time or increasing PA levels among participants. The studies predominantly used exploratory or pilot designs, with small sample sizes (range: 10‐32 participants) and short follow-up periods (≤12 wk), limiting the strength of the conclusions.

**Conclusions:**

Current evidence on the application of AI and machine vision in telephysiotherapy remains scarce and largely exploratory. Although the integration of wearable technology and ML algorithms shows technical feasibility and personalization potential, there is insufficient evidence to confirm improvements in PA outcomes. Future research should prioritize longitudinal clinical trials, ensure the inclusion of diverse populations, and evaluate behavioral outcomes to determine the real-world impact of these technologies in telerehabilitation.

## Introduction

Sedentary behavior is a major global health challenge. According to the World Health Organization (WHO), individuals who do not engage in adequate physical activity (PA) face a 20% to 30% higher risk of mortality compared to those who are regularly active [1]. Despite the well-documented health benefits of moderate-to-vigorous PA, large segments of the global population remain physically inactive [2]. In 1990, approximately 200 million adults worldwide lived with obesity; by 2022, this number had surged to 890 million, with the obesity rate more than doubling in every WHO region [3]. This rapid increase is closely linked to insufficient PA and sedentary lifestyle patterns [3]. Health guidelines recommend that adults engage in at least 150 minutes of moderate-intensity aerobic PA per week and perform strength-based exercises at least 2 times weekly [4]. Even light PA offers health benefits compared to none, and interruptions of sedentary behavior—such as taking breaks from sitting or incorporating movement into leisure time—are associated with improved health outcomes [5]. In Latvia, the situation mirrors global trends: only 9.5% of adults report being physically active for at least 30 minutes four or more times per week, and over 58% are classified as overweight or obese [4]. The growing burden of noncommunicable diseases, many of which are preventable through lifestyle changes, underscores the urgent need to develop effective, scalable, and accessible PA interventions [4]. Telerehabilitation has emerged as a promising strategy for delivering physiotherapy services remotely, thereby overcoming geographical and logistical barriers to care. Real-time telephysiotherapy has been shown to be as effective as traditional face-to-face rehabilitation in improving physical function and reducing musculoskeletal pain [2]. It also offers convenience, flexibility, and the potential for long-term engagement in health-promoting behaviors [2]. Simultaneously, digital health innovations such as artificial intelligence (AI), machine learning (ML), and machine vision technologies are gaining traction in health care and rehabilitation [2]. In this review, telephysiotherapy is defined as the remote delivery of physiotherapy-related assessment, monitoring, and intervention using digital technologies outside traditional clinical settings, most commonly in the home environment. Examples include video-based exercise supervision, wearable sensor–based activity monitoring, and AI-supported feedback systems designed to guide movement and promote PA.

These technologies enable personalized, real-time monitoring and feedback; improve data collection and analysis; and support the development of adaptive, behavior-informed interventions [2]. AI-driven tools are already being used for motion analysis, diagnostic support, and virtual rehabilitation, often in combination with wearable devices, mobile applications, or virtual reality systems [6]. However, despite the growing interest and potential, the specific application of AI and machine vision technologies in telephysiotherapy—particularly for the purpose of objectively monitoring PA and promoting daily movement in home environments—remains poorly defined. There is a lack of consolidated evidence on which digital tools are currently in use, what data they collect, how reliable and user-friendly they are, and whether they effectively change behavior or health outcomes in adult users [7]. To address existing research gaps resulting from limited consolidated knowledge in this field, this scoping review aimed to collect and systematize current evidence on the use of AI and machine vision in telephysiotherapy to promote PA and reduce sedentary behavior in healthy adult populations. While AI, ML, and machine vision technologies for PA monitoring are widely developed across multiple domains, including wearable computing and human-computer interaction, this review intentionally focuses on their application within telephysiotherapy contexts. This distinction is particularly relevant for intervention-focused research, where clinical relevance, remote supervision, and integration into physiotherapy workflows are essential. Consequently, the scope of evidence relevant to technology development (research questions 1 and 2) is broader than that of intervention studies (research question 3), which remain limited within telephysiotherapy settings.

To guide this objective, the following research questions were formulated:

- What AI and machine vision solutions are currently identified in telephysiotherapy?
- What are the characteristics of these technologies, including the types of data they monitor and their reliability?
- What AI and machine vision–based interventions have been used to promote daily PA, what are their features, which target populations have been studied, and what results have been reported?

## Methods

### Protocol and Registration

The review study was conducted from August 2024 to May 2025. This review was conducted in accordance with the Joanna Briggs Institute methodology for scoping reviews [8] and reported in accordance with PRISMA-ScR (Preferred Reporting Items for Systematic Reviews and Meta-Analyses Extension for Scoping Reviews) guidelines (Checklist 1) [9]. We followed the 6-stage framework for a scoping review proposed by Arksey and O’Malley [10]. The protocol was registered in the Open Science Framework [11].

The identified AI applications primarily focused on activity detection, movement quality assessment, and performance classification, with reported metrics such as classification accuracy and model precision. Only one study incorporated AI-driven personalized recommendations, highlighting that decision-support and behavior guidance systems remain underrepresented compared to monitoring-focused approaches. Only studies published in English were included due to resource constraints related to translation and to ensure accurate interpretation of methodological and technical details. While this approach may have excluded relevant studies published in other languages, English remains the dominant language of publication in AI, digital health, and rehabilitation research.

### Eligibility Criteria

Based on recommendations from the Joanna Briggs Institute [8] and the research questions to refine the focus of our scoping study and develop an effective search strategy, we defined the eligibility criteria for the PCC (population, concepts, and context) framework [8].

#### Population: Healthy Adults

This review focused on healthy adults aged 18 years and older who do not present with acute or chronic medical conditions. The decision to include this population was based on the need to evaluate preventive strategies aimed at promoting PA and reducing sedentary behavior in individuals without current health limitations. Healthy adults represent a critical target group for secondary prevention efforts, particularly as sedentary lifestyles are increasingly common in working-age populations and contribute to the long-term development of noncommunicable diseases [4]. In this review, “healthy adults” were defined as individuals without diagnosed acute or chronic physical diseases requiring medical treatment or structured rehabilitation. Individuals with lifestyle-related risk factors, such as overweight or obesity, were not excluded if no clinical diagnosis was reported. Mental health conditions were excluded only if they significantly affected physical functioning or participation in PA interventions.

#### Concept: AI, ML, and Machine Vision

The concept explored in this review includes AI, ML, and machine vision technologies used within telephysiotherapy contexts. AI refers to computational methods that enable systems to simulate human decision-making, adapt to data patterns, and provide real-time support [12]. ML, a subset of AI, allows for continuous system learning from large datasets, supporting personalized exercise recommendations and feedback loops [12]. Machine vision enables the interpretation of visual data for motion tracking, posture analysis, and automated exercise monitoring [12].

#### Context: Objective Remote Monitoring of Physical Activity (Telephysiotherapy)

The context of this review is remote physiotherapy—specifically, telephysiotherapy conducted outside formal health care settings, such as in the home environment. This includes digital interventions designed to objectively monitor or assess PA through sensors, video analysis, or algorithm-based platforms. These technologies are applied either for clinical assessment or as outcome measures in targeted interventions that aim to encourage sustained PA in daily life as part of secondary prevention strategies [8].

### Selection of Sources of Evidence

Individual studies published from January 2016 to April 2024 were considered for inclusion. The selection of sources for this scoping review was guided by clearly defined inclusion and exclusion criteria aligned with the study objectives and the PCC framework (Textbox 1). These criteria were established to ensure that the review captured relevant evidence on the application of AI, ML, and machine vision in telephysiotherapy aimed at promoting PA and reducing sedentary behavior in healthy adult populations. Given the rapid advancement of AI-based solutions in health care, a broad range of study types was included, provided they met clinical relevance and methodological transparency standards.

Textbox 1. **Inclusion and exclusion criteria.**

**Inclusion criteria**

- Population: healthy adults aged 18 years and older, without acute or chronic medical diagnoses;
- Concept: digital technologies incorporating artificial intelligence (AI), machine learning (ML), or machine vision applied in physiotherapy or physical activity (PA) monitoring
- Context: objective remote monitoring of PA conducted outside of health care institutions (eg, in the home environment), either for assessment or analysis or as an outcome measure in interventions
- Study type: primary research studies of any design (quantitative, qualitative, or mixed methods), including pilot and feasibility studies
- Publication status: published in a peer-reviewed journal
- Publication language: English
- Full-text availability
- Included keywords: artificial intelligence, machine learning, machine vision, telephysiotherapy, telerehabilitation, physical activity, exercise, wearable sensors, home-based rehabilitation, digital health, and remote monitoring

**Exclusion criteria**

- Population: individuals with acute or chronic medical conditions, pediatric or adolescent populations, and studies involving animal models
- Concept: digital technologies not related to AI, ML, or machine vision (eg, telemonitoring without intelligent data processing)
- Context: studies not involving PA as an outcome, or interventions not conducted remotely
- Setting: institutional or inpatient rehabilitation settings
- Study type: protocols, expert opinions, narrative or systematic reviews, editorials, letters, dissertations, or conference abstracts
- Publication status: non–peer-reviewed sources
- Publication language: languages other than English
- Full-text not available
- Excluded keywords: stroke, cancer, diabetes, depression, surgery, mice, rat, animal, in vitro, cognitive training, pharmacology, neurological disorder, and rehabilitation robotics

Studies focusing primarily on rehabilitation robotics were excluded a priori, as the review focused on software-driven AI and machine vision solutions applicable within telephysiotherapy contexts. Robotic systems often require specialized hardware, supervised environments, or clinical infrastructure, which limits their applicability to home-based, unsupervised telephysiotherapy for healthy adults.

### Information Sources

For this scoping review, peer-reviewed articles were searched in the following electronic databases: PubMed, ProQuest, Science Direct, and Wiley Online Library. These databases were selected to ensure broad coverage of biomedical, rehabilitation, and digital health literature relevant to the study’s PCC framework. The search results were imported into the Rayyan intelligent systematic review platform, where duplicates were identified and removed by multiple reviewers. A preliminary search was conducted between November 2024 and April 2025. The combined database search initially yielded 7253 references before deduplication. Only articles written in English were considered, and gray literature, including conference proceedings, dissertations, and preprints, was excluded from the final selection.

### Search Strategy and Search

Keywords were selected based on the defined PCC framework, as well as commonly used English terminology and synonyms. The keywords were grouped according to the PCC components and are outlined in Textbox 2.

Textbox 2. **Keyword combinations.**

- Population: healthy adults
- Concept: artificial intelligence, machine learning, and telephysiotherapy
- Context: objective remote monitoring of physical activity (telephysiotherapy) outside the health facility (home environment) can be used for assessment and analysis or as an outcome measure in a targeted intervention to promote daily physical activity as part of secondary prevention

The initial search strategy (Multimedia Appendix 1) was developed for PubMed in consultation with the Rīga Stradiņš University librarian and was later adapted for other databases. The Boolean logic used included:

AND between PCC components OR within each component’s synonyms

The following filters were used: language of publication (English), research on humans (English: human species), primary research, and publication date (from January 1, 2016). A search strategy was developed in the PubMed database using the following keywords:

Machine [All Fields] AND (“vision, ocular” [MeSH Terms]) OR “Machine Vision” [Text Word]) OR (“Artificial Intelligence/supply and distribution” [Majr] OR “Artificial Intelligence”[Text Word]) AND (“physical therapy modalities”[MeSH Terms] OR physiotherapy [Text Word]) OR (“Telerehabilitation” [Mesh] OR “Telerehabilitation” [Text Word]) OR (“Exercise” [Majr] OR “Physical Activity” [Text Word]) OR “sedentary behavior” [MeSH Terms] OR “Sedentary Behavior” [Text Word]

The search strategy used in the other databases included the following keywords:

(machine learning or artificial intelligence) AND (physical activity or exercise) OR sedentary behavior AND (telerehabilitation).

Database searches were conducted in PubMed, ProQuest, Science Direct, and Wiley Online Library from November 2024 to April 2025. The search results were imported into Rayyan, an intelligent systematic review platform. The study selection proceeded in 3 stages. First, titles and abstracts were screened to remove clearly irrelevant records. In the second phase, full-text studies were reviewed to assess eligibility based on inclusion and exclusion criteria and relevance to the primary and secondary research questions. Finally, a structured data extraction template was developed in Rayyan to support the systematic charting of evidence across studies. The template captured key details including participant characteristics (age, health status), the type of AI or machine vision technology used, PA monitoring methods, intervention design (if applicable), outcome variables, and main findings. Each study was reviewed independently by 2 team members. If Rayyan flagged a conflict between reviewers on inclusion or exclusion, a third reviewer made the final decision.

### Data Charting Process

This scoping review followed a structured approach to extract and analyze data from studies examining the application of AI, ML, and machine vision technologies in telephysiotherapy for promoting PA and reducing sedentary behavior among healthy adults. Data from the included studies were systematically charted using a standardized template to ensure consistency and relevance to the research objectives and the PCC framework.

Key variables extracted for each study included: paper ID, first author (name and surname), journal name, and title of the study to establish a bibliographic reference base. Geographic and demographic context was provided by documenting the country of the study, study aim, and participant characteristics (age, sex, and health status). To understand the methodological rigor and relevance of each study, information on study design (eg, feasibility study, pilot, experimental, or observational), applied methods, and analytical approaches was collected. Special emphasis was placed on detailing the digital technology used, specifically whether it involved AI, ML, or machine vision components, and how these technologies were implemented in the remote physiotherapy context.

Furthermore, studies were reviewed for their use of PA monitoring instruments (eg, wearables, video-based systems, and sensor technologies) and whether PA was measured as an intervention outcome, behavioral indicator, or clinical metric. For studies involving interventions, data were also collected on the structure and duration of the intervention, feedback mechanisms, and whether the systems were tailored to behavior change principles.

Given the broad diversity in how AI and digital physiotherapy interventions are designed and evaluated, studies varied in their terminology and framing of participants and settings (eg, “healthy volunteers,” “nonclinical adults,” and “remote physiotherapy users”). This heterogeneity was noted to better understand the generalizability and scalability of current AI-enabled telephysiotherapy solutions for healthy adult populations. The data charting process facilitated the synthesis of current practices, innovation trends, and evidence gaps in using AI-based technologies to support remote PA interventions. All data were independently extracted by 2 reviewers and cross-checked for accuracy and completeness. Discrepancies were resolved through team discussion to maintain methodological rigor and transparency.

### Synthesis of Results

Following the final stages of the 6-stage framework for scoping reviews proposed by Arksey and O’Malley [10], after identifying the research questions, locating relevant studies, selecting eligible sources, and charting the data, the findings were collated, summarized, and reported. First, a *descriptive numerical summary* was conducted to outline the number of included studies, publication years, countries of origin, study designs, and types of digital technologies investigated. This provided a broad overview of the research landscape and highlighted the diversity of approaches across different geographic and methodological contexts. Second, a *qualitative thematic analysis* was applied to extract key themes related to the use of AI, ML, and machine vision technologies in remote PA monitoring and intervention delivery. This thematic synthesis focused on technology types, behavioral targets, implementation contexts, feedback mechanisms, and outcome measures. Third, *research findings were narratively evaluated* to identify results aligned with the study’s core research questions. These included the types and features of digital solutions used, the contexts in which they were implemented, and their impact on promoting PA and reducing sedentary behavior. Finally, the *interpretation of findings* was contextualized in relation to practical implications for telephysiotherapy practice, limitations in current evidence, and recommendations for future research and policy development.

Results were organized descriptively and are presented using structured tables, accompanied by narrative explanations to clarify their relevance and significance.

## Results

### Selection of Sources of Evidence

To improve clarity and synthesis, the results are organized thematically rather than as isolated study summaries. The findings are presented according to three overarching themes: (1) types of AI and machine vision technologies used, (2) data sources and performance characteristics, and (3) intervention-related features and outcomes.

Our search identified a total of 7253 records from 4 electronic databases: PubMed, ProQuest, Science Direct, and Wiley Online Library. Among these, 1979 duplicate records were identified and removed, resulting in 5274 records eligible for title and abstract screening, indicating the effectiveness and feasibility of the search strategy. Following the initial screening based on predefined inclusion and exclusion criteria, 5210 records were excluded due to reasons such as irrelevant population (eg, clinical samples), concept mismatch (eg, digital tools without AI or machine vision), or nontelephysiotherapy contexts. Sixty-four full-text studies were assessed for eligibility. Of the 64 full-text studies reviewed, 60 were excluded for the following reasons: inclusion of clinical populations with chronic or acute diagnoses, absence of AI or machine vision components, focus on pediatric or adolescent populations, use of robotics outside telephysiotherapy, and nonprimary study designs such as reviews, conference abstracts, or protocols. A total of 4 studies met all inclusion criteria and were included in this scoping review. The selection process is illustrated in the PRISMA (Preferred Reporting Items for Systematic Reviews and Meta-Analyses) flow diagram (Figure 1).

**Figure 1.**
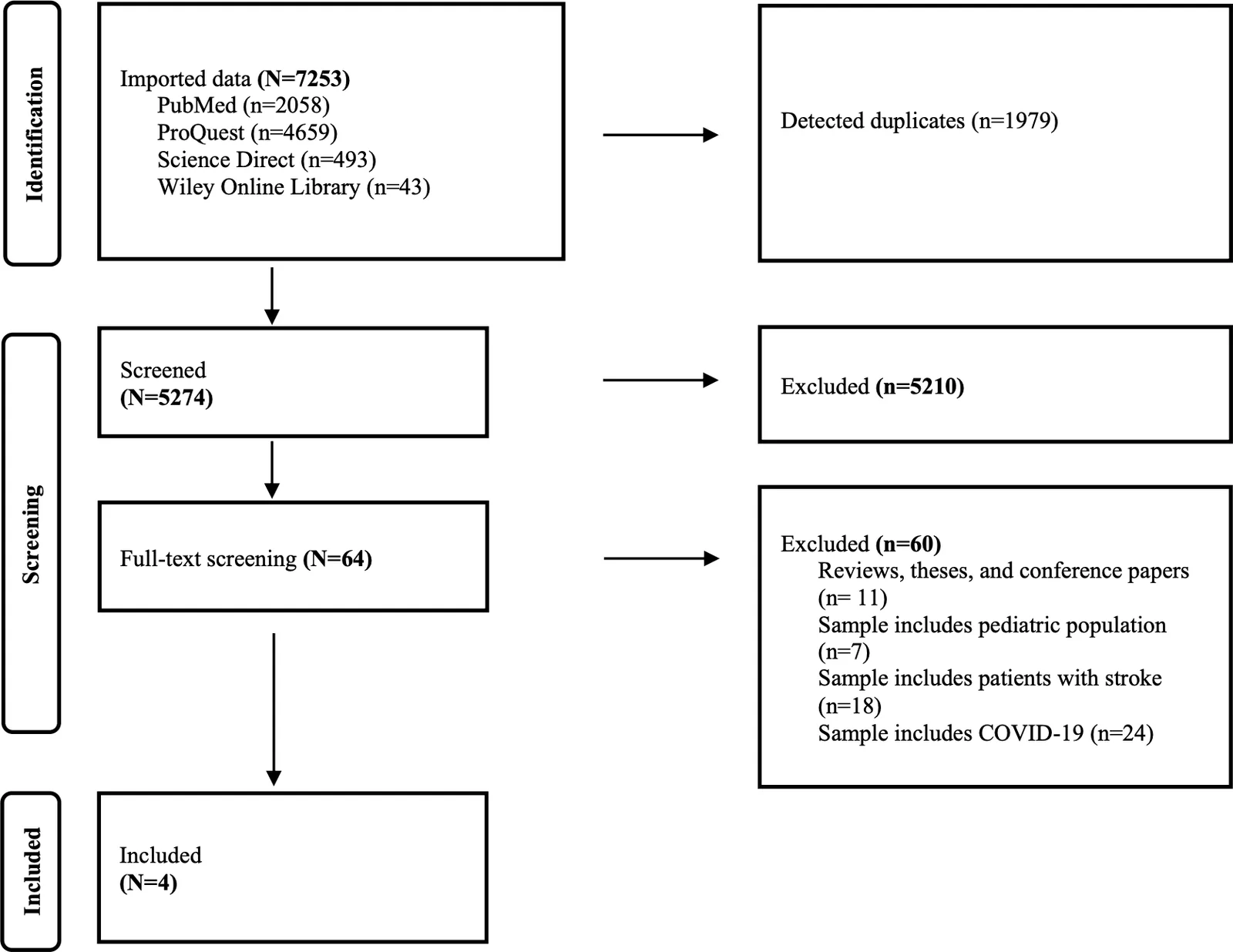
PRISMA (Preferred Reporting Items for Systematic Reviews and Meta-Analyses) flow diagram for the selection of sources of evidence.

### Characteristics of Included Studies

The characteristics of the 4 selected studies in this review are summarized in Figure 1. Given that AI, ML, and machine vision were among the inclusion criteria, all included studies incorporated at least one method for the objective assessment of PA.

The identified technologies—their types and the monitored data in the analyzed studies—are summarized in Table 1.

**Table 1.** Characteristics of included studies for analysis.

Author, year (country)	Research design	Participants	Intervention	Artificial intelligence and machine vision technologies	Summary of main results
Du et al [13], 2022 (United States)	Experimental comparative validation with a preprepared public dataset UI-PRMD^a^	n=21 healthy adults (age and gender details not specified)	Developed a deep learning framework with GCNs^b^ for physical activity assessment. Includes video-level and frame-level CoP^c^ parameter assessment.	GCN, ST-GCN^d^, and adaptive GCN 3D body landmarks from video (with OpenPose) Personalized CoP indicator assessment from video, also using only an RGB camera (eg, smartphone)	The proposed method with GCN effectively estimates CoP path length, sway area, and position (as balance indicators). The multitask model showed higher accuracy compared to single-task models. A new dataset with synchronized 3D pose and CoP data was created and published. The study confirms that video analysis can replace expensive laboratory equipment in everyday balance and rehabilitation monitoring.
Chatterjee et al, 2022 [12] (Norway)	Experimental study with MM algorithm testing on public and private data	Public dataset (Fitbit): n=33 adults Private dataset (MOX2-5 sensor): n=16 adults (aged 18‐64 y), 30 days of observation in healthy adults	Created a prototype of the eCoach system for generating personalized activity recommendations. Combined ML^e^ algorithms (eg, decision tree, SVM^f^, and random forest) with a semantic ontology. Used transfer learning and incremental learning approaches to improve and personalize models.	ML classifiers: decision tree, random forest, SVM, and KNN^g^. Use of ontologies for semantic data structuring and personalization. Data from Fitbit and medical-grade sensor MOX2-5.	Decision tree classifier with the “entropy” indicator showed the highest accuracy: Fitbit data: 97.5% accuracy MOX2-5 data: 96.1% accuracy. After transfer learning: 97.99% accuracy. The eCoach system can provide personalized recommendations based on activity data and user preferences. The ontology model improves the explainability of recommendation logic (through SPARQL^h^, OWL^i^, and RDF^j^).
Boyer et al [14], 2023 (Canada)	Experimental research with sensor data collection and MM model development	Twenty one participants, age: 18‐65 years Health status: healthy volunteers without musculoskeletal disorders Gender: 8 men and 12 women	Participants performed 4 different physical therapy exercises (eg, straight leg raises and knee bends) while wearing an IMU^k^ sensor (MPU-9250) attached to their ankle. The sensor collected accelerometer, gyroscope, and magnetometer data during the exercises.	IMU sensors as a data source (acceleration, rotation, and magnetic field measurements). ML algorithms for exercise quality recognition and monitoring. Classification of exercises as “correct” versus “incorrect.” The approach allows remote monitoring of patient self-discipline and technique.	ML models can accurately determine whether a participant is performing the intended physical activity or not. The best models achieved up to 95% accuracy, indicating that this approach is promising for remote patient monitoring and motivation during the rehabilitation process. Thus, such systems can improve patient compliance and provide objective monitoring without the direct presence of a therapist.
Mennella et al [15], 2023 (Italy)	Experimental development with algorithm development and testing	Seventeen healthy participants (9 women and 8 men) with an average age of 25.6 (SD 6.4) years took part in the experiment	Motion data were obtained using MoveNet, which generates 3D skeleton coordinates. A lightweight deep neural network with a single hidden layer was developed to classify both ROM^l^ (3 classes: low, normal, and high) and compensation (correct or compensatory execution). ROM values calculated as angles between joints were used for classification.	ML algorithm XGBoost^m^ was used to classify whether a participant performed a movement correctly or with compensation. The data were obtained using IMU sensors that recorded the angular velocity of 3 body segments: the thoracic spine, lumbar spine, and pelvis. Time-domain features were extracted from the data, such as mean value, maximum, RMS^n^, and so on.	ROM classification: average accuracy of 89% achieved (ranging from 81%-95% depending on the exercise). Compensatory movement classification: average accuracy of 98%. Compared to manual classification by clinicians, the system’s performance is very similar or even more accurate, thus improving objectivity and repeatability.

a UI-PRMD: University of Idaho – Physical Rehabilitation Movement Data.

b GCN: graph convolutional network.

c CoP: center of pressure.

d ST-GCN: spatial-temporal graph convolutional networks.

e ML: machine learning.

f SVM: support vector machine.

g KNN: k-nearest neighbor.

h SPARQL: is a query language used to search and change data stored as graphs on the web.

i OWL: object windows library.

j RDF: resource description framework.

k IMU: inertial measurement unit.

l ROM: range of motion.

m XGBoost: extreme gradient boosting.

n RMS: root mean square.

In 2 of the 4 experimental studies, a body-mounted inertial measurement unit sensor was identified as the data source, and ML algorithms were used to recognize and monitor exercise quality [14].

### AI and Machine Vision Solutions Currently Identified in Telephysiotherapy

A deep learning framework was developed with graph convolutional networks for PA assessment [13]. Video-based methods were used to assess center-of-pressure (CoP) parameters at both the level of the whole body and individual body segments [13]. An eCoach prototype system was designed to generate personalized activity recommendations [12]. ML algorithms (eg, decision tree, support vector machine, and random forest) were integrated with semantic ontology [14]. ML algorithms were applied for exercise quality recognition and monitoring [13]. The extreme gradient boosting ML algorithm was used [16].

### AI and Machine Vision–Based Interventions

One of the studies included in this review introduces a system that allows real-time quantitative assessment of motor patterns in rehabilitation exercises using 2D video analysis. The main objective is to discreetly observe the exercises performed by a person during a therapy session and assess whether they are being performed correctly or incorrectly according to the instructions. The assessment corresponds to the assessment of range of motion and compensatory movement patterns that the participant may perform during a rehabilitation session [15].

To assess a patient’s physical performance using 2D video, the methodology consists of the following steps: body reference point extraction, data normalization, and classification. A network approach was investigated to classify correctly performed exercises. The classifier must be sufficiently stable to assign a label to each frame that indicates incorrect execution and good movement quality (normal movement patterns without erroneous movement categories, such as limited range of motion or compensatory movements) [15].

MoveNet is an upward-looking estimation model that uses heat maps to locate key points on a person’s body. The model is designed to run in a browser using TensorFlow.js or on devices using TensorFlow Lite in real time. The MoveNet Thunder variant was chosen, which provides greater power and better prediction quality while maintaining high real-time speed [16]. The architecture of the MoveNet Thunder version uses an RGB video frame or image with a resolution of 256×256 as input [15].

The research team compiled a dataset of shoulder physiotherapy exercises. It includes both concentric and isometric exercises, 9-axis data (accelerometer, gyroscope, and magnetometer data), as well as data from nonexercise activities (ie, out-of-distribution data) [14]. The researchers used a combination of ML algorithms to analyze the data and identify the most effective shoulder exercises [14].

A semantic ontology model was created to annotate ML prediction results and personal preferences, conceptualizing personalized recommendation generation using a hybrid approach. The study used a transfer learning approach to improve ML model training and performance, as well as a gradual learning approach to process the daily increasing volume of data and adapt it to ML models [12].

“Deep learning–based video analysis for human performance evaluation” investigated 2 typical tasks: a clinical biometric evaluation task—the assessment of the CoP metric—and a performance evaluation task—the assessment of physical rehabilitation exercises. The CoP metric and physical rehabilitation exercise assessment aimed to develop a new video analysis system for human performance assessment. Two gold-standard CoP indicators—CoP path length, which reflects the total distance or length of the CoP trajectory while standing, and CoP oscillation area, an ellipse that covers a certain percentage of the CoP trajectory—are widely used to quantitatively assess posture control. A shorter CoP path length and oscillation area usually indicate better postural control, while a longer length is associated with aging, diseases, or injuries that impair balance control or mobility [13].

## Discussion

### Principal Results

This scoping review aimed to collect and systematize current evidence on the use of AI and machine vision in telephysiotherapy to promote PA and reduce sedentary behavior in healthy adult populations. Only 4 studies met the eligibility criteria, highlighting the limited but growing body of research in this emerging field. The findings provide insight into the types of digital technologies currently in use, their core features, application settings, and the methodological gaps that need to be addressed in future research. The identified studies used a variety of digital approaches to support remote physiotherapy interventions. These included ML algorithms such as extreme gradient boosting and hybrid ensemble classifiers used to analyze sensor-derived movement data [13], as well as deep learning models like graph convolutional networks designed to interpret biomechanical features [13]. One study used a semantic ontology to structure and personalize PA recommendations [12], while another applied video-based systems to calculate the CoP using a standard RGB camera in a home environment [16]. These solutions aimed to support PA monitoring and personalized feedback delivery, aligning with the broader goal of increasing movement and reducing sedentary behavior outside traditional clinical settings [16]. In relation to the review’s research questions, the first objective was to identify AI and machine vision solutions currently applied in telephysiotherapy. The review revealed that these technologies are predominantly in the developmental or pilot-testing stages and are often integrated into wearable sensor platforms or computer vision–based applications [17]. Their use focused on either real-time exercise monitoring or movement analysis, reflecting a technical interest in enhancing the precision of remote physiotherapy systems [17]. The second objective addressed the characteristics of these technologies, including the type of data monitored and the reliability of the systems. While most studies demonstrated that it is technically feasible to implement AI and machine vision in home-based physiotherapy contexts, they rarely included robust evaluations of system accuracy, user engagement, or long-term adherence [17]. In many cases, technical functionality was prioritized over behavioral or clinical outcomes, indicating a gap between innovation and implementation readiness [16,18]. The third research question focused on identifying specific interventions and their outcomes. Although each study aimed to encourage PA, none reported statistically significant improvements in PA levels or reductions in sedentary behavior [12-15]. This may be explained by several factors, including small sample sizes, short intervention durations, and the exploratory nature of the study designs [12-15]. These limitations underscore the need for more rigorous evaluation methods and stronger behavioral outcome tracking in future research [19]. It is important to note that the limited number of included studies does not reflect the maturity of PA recognition technologies themselves, but rather the early stage of their translation into telephysiotherapy interventions for healthy adults. While AI-based activity monitoring methods are well established in broader digital health and human movement research, their integration into structured, behavior-oriented telephysiotherapy programs remains sparse.

### Implications for Practice

The findings of this review emphasize the potential of integrating innovative digital solutions—specifically those using AI and machine vision—into telephysiotherapy practice aimed at healthy adults. These technologies offer opportunities to enhance remote PA monitoring through automated feedback, personalized recommendations, and real-time performance analysis [14,15]. For physiotherapists, this means the possibility of expanding their role beyond in-clinic care to continuous, preventive support in home and community settings. By leveraging AI-enabled systems, clinicians could more effectively promote PA adherence, reduce sedentary behavior, and support early intervention for individuals at risk of inactivity-related health issues. However, the limited number of studies and variation in approaches also highlight the need for a more standardized framework in designing and implementing AI-based PA monitoring. There is currently no consistent methodology for selecting sensors, interpreting data, or integrating intelligent systems into physiotherapy protocols. This inconsistency hinders comparability between studies and limits the transferability of findings to real-world practice [14]. Just as prior reviews in clinical populations have called for harmonized guidelines on accelerometry and self-reported PA [17], similar efforts are needed in the context of healthy adult telephysiotherapy. Additionally, most systems evaluated in the included studies were still in early development stages, lacking validation in routine practice. For digital health technologies to be integrated meaningfully into physiotherapy, future efforts must ensure not only technical feasibility but also clinical relevance, user-centered design, and regulatory compliance. This includes establishing protocols for data accuracy, privacy, and therapist oversight when using automated assessment and feedback tools [7].

### Limitations

While the review highlighted technical innovation and promising concepts, it also exposed several limitations in the current literature. The scope of most studies was narrow, with limited demographic diversity and small participant numbers [12-15]. Longitudinal data were lacking, and many systems were only tested in highly controlled or simulated environments [14,15]. As a result, generalizability remains limited, and the effectiveness of these technologies in real-world settings is still largely unknown [13].

Another important limitation of this review is the exclusion of computer science–oriented publication venues, particularly ACM journals and conference proceedings (eg, Interactive, Mobile, Wearable and Ubiquitous Technologies), which frequently report state-of-the-art advances in PA recognition and wearable sensing. As this review prioritized clinically oriented, peer-reviewed health databases, some advanced algorithmic developments may not be represented. Future interdisciplinary reviews should integrate biomedical and human-computer interaction literature to provide a more comprehensive overview of technological innovation and readiness.

The selection of databases represents another limitation of this review. While PubMed, ProQuest, ScienceDirect, and Wiley Online Library were chosen to ensure coverage of biomedical and rehabilitation-focused literature, additional databases such as CINAHL, Embase, IEEE Xplore, Web of Science, and Scopus may have captured a broader range of AI and machine vision–related studies. The restricted database selection, combined with other eligibility criteria, likely contributed to the small number of included studies and should be considered when interpreting the scope of the findings.

### Comparison With Prior Work

The findings of this scoping review partially align with previous research in adjacent areas such as telerehabilitation, digital physiotherapy, and AI-supported movement analysis. Prior systematic reviews have highlighted the increasing integration of AI and ML into rehabilitation technologies, particularly for clinical populations such as patients recovering from stroke, orthopedic surgery, or neurological conditions [18]. However, in contrast to those studies, this review focused specifically on healthy adults as a target group for secondary prevention and behavior change, rather than posttreatment rehabilitation. Previous literature has also noted the effectiveness of telehealth platforms in improving access to care, enabling remote monitoring, and supporting adherence to rehabilitation protocols [20]. However, most of these platforms rely on conventional video calls or basic sensor data, without the incorporation of intelligent systems capable of processing complex movement patterns in real time. This review identified a small number of studies that went beyond traditional telephysiotherapy, using AI or machine vision to automate analysis, personalize feedback, or predict PA levels. These applications, though promising, are still relatively novel and not yet widely implemented or evaluated at scale. Moreover, the lack of statistically significant outcomes in the reviewed studies contrasts with findings from other digital health research, where AI-supported interventions have demonstrated improvements in chronic disease management or physical function [21]. This discrepancy may be explained by differences in study design, participant populations, and the early-stage development of many AI and machine vision tools in the PA domain. The review also supports earlier conclusions that human-technology interaction remains a critical factor in the success of digital interventions [21]. While some prior work has emphasized the potential of gamification or virtual coaching to boost engagement, the studies included in this review rarely addressed motivational strategies or user experience design, underscoring a persistent gap between technical capabilities and behavioral outcomes [12].

Despite the high reported classification accuracy in controlled or semicontrolled settings, the reviewed studies provide limited evidence regarding the robustness of AI and machine vision systems in real-world home environments. Practical challenges, such as variable lighting conditions, occlusion, sensor placement inconsistency, limited camera viewpoints, and user adherence, were rarely addressed. Moreover, high technical performance did not translate into measurable reductions in sedentary behavior or sustained increases in PA, indicating a gap between algorithmic feasibility and behavioral effectiveness.

### Future Directions

Despite the high reported classification accuracy in controlled or semicontrolled settings, the reviewed studies provide limited evidence regarding the robustness of AI and machine vision systems in real-world home environments. Practical challenges such as variable lighting conditions, occlusion, sensor placement inconsistency, limited camera viewpoints, and user adherence were rarely addressed. Moreover, high technical performance did not translate into measurable reductions in sedentary behavior or sustained increases in PA, indicating a gap between algorithmic feasibility and behavioral effectiveness.

The findings of this scoping review suggest that future research should prioritize investigating how AI and machine vision technologies can not only monitor but also actively promote PA participation in healthy adult populations. While several studies have demonstrated the technical feasibility of these tools, limited research has examined their direct impact on actual behavior change, such as increased daily PA or reduced sedentary time [5]. Further exploration is needed into the integration of intelligent, interactive features—such as real-time feedback, adaptive difficulty, or personalized goal setting—within telephysiotherapy platforms. These elements have the potential to increase user engagement, support long-term adherence, and enhance motivation through continuous remote guidance [2]. As seen in adjacent fields, systems incorporating behavioral science and digital interaction strategies, including gamification or computer vision–based exercise feedback, may be more effective than passive monitoring alone [7]. It is also important that future studies evaluate user perspectives, including acceptance, usability, and trust in AI-driven physiotherapy tools. Understanding how individuals engage with and respond to these technologies in real-world settings is essential for designing interventions that are not only technically robust but also meaningful and sustainable [22]. In parallel, research should address clinical integration pathways, particularly the role of physiotherapists in overseeing or comanaging AI-supported remote programs. Moreover, the review identified a lack of standardized protocols for digital PA assessment in the context of AI and telephysiotherapy. Developing unified guidelines for system design, data interpretation, and outcome evaluation would improve comparability across studies and support evidence-based implementation into clinical and preventive care frameworks [15].

### Conclusions

This scoping review examined the current evidence on the use of AI and machine vision in telephysiotherapy to promote PA and reduce sedentary behavior among healthy adults. Although the findings demonstrated the technical feasibility of integrating intelligent systems into remote physiotherapy, the number of eligible studies was limited, and most were early-stage feasibility or pilot trials. While these technologies showed potential for personalized feedback, automated monitoring, and behavior support, there was insufficient evidence on their long-term effectiveness or impact on PA outcomes. The review highlights the need for more rigorous research involving diverse populations, standardized assessment protocols, and real-world implementation. With further development and validation, AI and machine vision could offer valuable tools to enhance preventive physiotherapy, support active lifestyles, and improve accessibility to personalized care beyond traditional clinical settings.

## Supplementary material

10.2196/80459 Multimedia Appendix 1 Full search strategies.

10.2196/80459 Checklist 1 PRISMA-ScR checklist.

## Abbreviations

AI: artificial intelligence
CoP: center of pressure
ML: machine learning
PA: physical activity
PCC: population, concepts, and context
PRISMA: Preferred Reporting Items for Systematic Reviews and Meta-Analyses
PRISMA-ScR: Preferred Reporting Items for Systematic Reviews and Meta-Analyses Extension for Scoping Reviews
WHO: World Health Organization
