# Supplementary material for: Leveraging AI and Machine Vision in Telephysiotherapy for Promoting Physical Activity and Reducing Sedentary Behavior: Scoping Review
Source: JMIR Rehabil Assist Technol. 2026 Oct 15;13:e80459. doi: 10.2196/80459 (PMC13671600; doi:10.2196/80459)
Supplement: Supplementary file 2 [file rehab-v13-e80459-s001.docx]

Appendix 1. Full Search Strategies

| **Database** | **Search terms** |
| --- | --- |
| MEDLINE  (Pubmed) | ("Artificial Intelligence"[Mesh] OR "Machine Learning"[Mesh] OR "Artificial Intelligence"[tiab] OR "Machine learning"[tiab] OR "Machine vision"[tiab] OR "Computer vision"[tiab])  AND ("Telerehabilitation"[Mesh] OR telephysiotherapy[tiab] OR telerehabilitation[tiab] OR "remote physiotherapy"[tiab])  AND ("Physical Activity"[Mesh] OR "Exercise"[Mesh] OR "physical activity"[tiab] OR exercise[tiab] OR "sedentary behavior"[Mesh] OR "sedentary behaviour"[tiab])  AND (adult[Mesh] OR adults[tiab] OR "healthy adults"[tiab]) |
| ProQuest | ("artificial intelligence" OR "machine learning" OR "machine vision" OR "computer vision")  AND (telephysiotherapy OR telerehabilitation OR "remote physiotherapy")  AND ("physical activity" OR exercise OR "sedentary behavior" OR "sedentary behaviour")  AND (adult* OR "healthy adults") |
| ScienceDirect | ("artificial intelligence" OR "machine learning" OR "machine vision" OR "computer vision") AND (telephysiotherapy OR telerehabilitation OR "remote physiotherapy") AND ("physical activity" OR exercise OR "sedentary behavior" OR "sedentary behaviour") AND (adult OR adults OR "healthy adults") |
| Wiley Online Library | ("artificial intelligence" OR "machine learning" OR "machine vision" OR "computer vision")  AND (telephysiotherapy OR telerehabilitation OR "remote physiotherapy")  AND ("physical activity" OR exercise OR "sedentary behavior" OR "sedentary behaviour")  AND (adult* OR "healthy adults") |
